# The Evolving Landscape of Chronic Lymphocytic Leukemia on Diagnosis, Prognosis and Treatment

**DOI:** 10.3390/diagnostics11050853

**Published:** 2021-05-10

**Authors:** Claudia Pérez-Carretero, Isabel González-Gascón-y-Marín, Ana E. Rodríguez-Vicente, Miguel Quijada-Álamo, José-Ángel Hernández-Rivas, María Hernández-Sánchez, Jesús María Hernández-Rivas

**Affiliations:** 1 Cancer Research Center (IBMCC) CSIC-University of Salamanca, 37007 Salamanca, Spain; claupeca@usal.es (C.P.-C.); anita82@usal.es (A.E.R.-V.); mquijada@usal.es (M.Q.-Á.); 2 Instituto de Investigación Biomédica (IBSAL), 37007 Salamanca, Spain; 3 Department of Hematology, University Hospital of Salamanca, 37007 Salamanca, Spain; 4 Department of Hematology, Infanta Leonor University Hospital, 28031 Madrid, Spain; igonzalezg@salud.madrid.org (I.G.-G.-y-M.); jahernandezr@salud.madrid.org (J.-Á.H.-R.); 5 Department of Medicine, Complutense University, 28040 Madrid, Spain; 6 Department of Medicine, University of Salamanca, 37008 Salamanca, Spain

**Keywords:** chronic lymphocytic leukemia (CLL), diagnosis, prognosis, treatment, evolution, state-of-the-art

## Abstract

The knowledge of chronic lymphocytic leukemia (CLL) has progressively deepened during the last forty years. Research activities and clinical studies have been remarkably fruitful in novel findings elucidating multiple aspects of the pathogenesis of the disease, improving CLL diagnosis, prognosis and treatment. Whereas the diagnostic criteria for CLL have not substantially changed over time, prognostication has experienced an expansion with the identification of new biological and genetic biomarkers. Thanks to next-generation sequencing (NGS), an unprecedented number of gene mutations were identified with potential prognostic and predictive value in the 2010s, although significant work on their validation is still required before they can be used in a routine clinical setting. In terms of treatment, there has been an impressive explosion of new approaches based on targeted therapies for CLL patients during the last decade. In this current chemotherapy-free era, BCR and BCL2 inhibitors have changed the management of CLL patients and clearly improved their prognosis and quality of life. In this review, we provide an overview of these novel advances, as well as point out questions that should be further addressed to continue improving the outcomes of patients.

## 1. Introduction

Chronic lymphocytic leukemia (CLL) is the most common form of adult leukemia in the USA and in Europe, with an incidence of approximately 4.2 cases per 100,000 people per year [1]. Its median age at diagnosis ranges from 70 to 72 years, with a male predominance of roughly 2:1 cases [2,3]. The incidence increases with age, and the prevalence is likely to increase due to demographic changes in society in the near future. CLL is a clonal B-cell lymphoproliferative disorder characterized by the accumulation of small, mature, CD5+CD23+ neoplastic lymphocytes in the peripheral blood, bone marrow, spleen and other lymphoid tissues [4,5]. It displays remarkable clinical heterogeneity, ranging from an indolent disease with no requirement for treatment in some patients to rapid disease progression and subsequent treatment refractoriness in others [6]. Taking into account that the majority of patients do not require treatment at diagnosis but, rather, a ‘watch and wait’ strategy, the first main aim is to accurately assess the risk of developing a progressive disease in need of therapy at a given time point [2,7].

CLL development is preceded by monoclonal B cell lymphocytosis (MBL), a premalignant state defined by the presence of less than 5 × 10^9^/L clonal B cells in the absence of lymphadenopathy, organomegaly or cytopenias [8]. At the other end of the spectrum, CLL may undergo histologic transformation into an aggressive B-cell lymphoma (commonly diffused large B-cell lymphoma or Hodgkin’s lymphoma). This process is termed Richter’s transformation and is associated with a very dismal clinical outcome [9].

The high prevalence of this type of leukemia and the vast availability of tumor cells in the peripheral blood of these patients has historically placed CLL at the forefront of cancer genetic discovery. The implementation of cutting-edge genomic technologies into the study of CLL has extremely refined the prognosis of this disease overtime: from the discovery of recurrent chromosomal abnormalities by a chromosome banding analysis (CBA) or fluorescence in situ hybridization (FISH) to the explosion of high-throughput sequencing techniques for the detection of driver mutations with clinical implications [10,11,12,13,14,15]. In parallel, the study of the biological processes underlying CLL pathogenesis has profoundly changed the treatment landscape of this disease, leading to the striking development of targeted therapies such as BCR signaling or BCL2 inhibitors, almost completely displacing chemotherapy-based regimens from the treatment algorithms nowadays [3,16]. In this review, we will cover the evolving process by which these genetic and biology discoveries have shaped the diagnosis, prognosis and treatment of CLL over the last few decades.

## 2. Diagnosis

The diagnostic criteria for CLL, although refined over time, have not dramatically changed since the first guidelines stablished in the 1990s [17,18]. In 2008, the International Workshop on Chronic Lymphocytic Leukemia (iwCLL) published consensus guidelines with updated recommendations for the management of CLL in general practice [19]. This version was updated with minor modifications in terms of the diagnostic criteria in 2018 [20]. Nowadays, the diagnosis of CLL is mainly based on laboratory features, namely blood count, morphology and immunophenotyping [1,20].

CLL is first suspected when an absolute peripheral lymphocytosis of 5 × 10^9^/L clonal B cells is found in the peripheral blood [17]. This lymphocytosis must persist for longer than 3 months, according to the latest version of iwCLL guidelines [20]. The presence of a cytopenia caused by clonal bone marrow involvement establishes the diagnosis of CLL regardless of the peripheral B-lymphocyte count [20]. Bone marrow aspirate and biopsy are not required for the diagnosis of CLL. However, if done, the marrow often demonstrates >30% lymphocytes [21].

The leukemia cells in the blood smear are characteristically small, mature lymphocytes with a narrow border of cytoplasm and a dense nucleus lacking discernible nucleoli and having partially aggregated chromatin. Large atypical cells, cleaved cells and prolymphocytes are also often seen on the peripheral smear and may account for up to 55% of the peripheral lymphocytes [17,22].

The clonality of the peripheral circulating B-lymphocytes needs to be confirmed by flow cytometry. Based on the antigenic profile, Matutes et al. designed in 1994 an immunologic score system (Matutes score, MS) to ensure the diagnosis of CLL [23]. In this scoring system, a value of 0 or 1 was given according to the expression of the five markers of CD5, CD23, FMC7, surface immunoglobulin M (sIgM) and CD22. Most CLL cases had a score of 4 or 5, whereas non-CLL cases had a score of less than 4. It was shown subsequently that CD22 could be advantageously replaced by CD79b [24]. The scoring proposed in the modified MS has been the basis of diagnosis for the following years and was defined by a strong expression of CD5 (normally expressed on T cells) and CD23, a low or absent expression of CD79b, sIgM and FMC7 [24]. However, in some cases, differential diagnosis on the basis of the markers included in this score has been challenging due to some limitations affecting reproducibility—in particular, flexibility in marker expression.

Other potentially informative markers have been evaluated to be considered for the CLL diagnosis, although a consensus concerning these novel markers has not been reached yet [25,26,27,28,29]. In 2018, a recent large harmonization effort confirmed that a panel of CD19, CD5, CD20, CD23 and sIg kappa or lambda is usually sufficient to establish the diagnosis of CLL using peripheral blood samples [30]. In borderline cases, markers such as CD43, CD79b, CD81, CD200, CD10 or ROR1 may help to refine the diagnosis [30]. The current criteria for the CLL diagnosis have been updated by the iwCLL, the World Health Organization (WHO) and the European Research Initiative on CLL (ERIC) [8,20,30,31]. However, MS is still used in many centers. A lymphoid node biopsy and/or bone marrow biopsy may be helpful if immunophenotyping is not conclusive for the diagnosis of CLL [2].

The 2008 WHO classification included CLL, together with small lymphocytic lymphoma (SLL), as mature B-cell neoplasms entities [32]. SLL is characterized by the presence of fewer than 5 × 10^9^/L lymphocytes with lymphadenopathy and without cytopenias, although this diagnosis should be confirmed by a lymph node biopsy.

The 2008 WHO classification of lymphoid neoplasms also defined MBL when the presence of less than 5 × 10^9^/L clonal B lymphocytes happened in the absence of lymphadenopathy or organomegaly (as defined by a physical examination or CT scan), cytopenias or disease-related symptoms [32,33,34,35]. About 1% to 2% of MBL cases progress to CLL per year [36]. In the 2016 update, the WHO differentiated “low-count MBL” from “high-count MBL” according to the size of the monoclonal B-cell population (cutoff: 0.5 × 10^9^/L) [8].

As previously mentioned, CLL can develop Richter Syndrome (RS), a secondary and aggressive lymphoma with an incidence rate of ~0.5% per year. Most cases of RS (95%) consist of a histologic transformation to diffuse large B-cell lymphoma (DLBCL) and, less often, Hodgkin’s lymphoma (HL) [37]. Commonly, RS clinically presents with rapidly enlarging lymph nodes, accompanied by the presence of constitutional symptoms, fever and weight loss, together with elevated lactate dehydrogenase (LDH) levels, when lymph nodes enlarge rapidly [9,38]. A lymph node biopsy is required to establish the diagnosis of a transformation into an aggressive lymphoma [19]. DLBCL-RS is clonally related to the underlying CLL in more than 80% of cases and has a worse outcome than clonally unrelated cases, which have a prognosis similar to de novo DLBCL [39].

Once the diagnosis of CLL is confirmed, patients should undergo additional laboratory evaluations to help the physician predict the prognosis and guide the treatment approach. Very recently, the ESMO Clinical Practice Guidelines provided recommendations on the management of CLL for diagnosis, treatment and follow-up [2].

## 3. Prognosis

### 3.1. Evolution of Prognostic Factors over the Last Decades

CLL has long been known to be an extremely clinically heterogeneous disease [5,6,40] that can be linked by the vast genetic heterogeneity observed in patients through optimal sequencing strategies [14,41]. In the last decades, the improved understanding of CLL pathogenesis has resulted into the identification of a great number of prognostic markers (clinical systems, serum markers, genetic alterations, etc.), significantly improving patient stratification [42]. With the advent of targeted agents (TA), the value of some of them is in question. For this reason, prognostication in CLL remains an active research field in order to define not only the prognostic markers able to predict the clinical course at diagnosis but, also, the predictive markers able to predict the response to treatment in the era of targeted therapies. Table 1 shows the main markers with clinical significance in terms of prognosis.

### 3.2. Prognostic Markers

#### 3.2.1. Rai and Binet Staging Systems

Classic Rai and Binet staging systems were established more than 40 years ago due to the need to classify CLL patients with different outcomes [43,44]. They are based on the clinical parameters and still remain the first approach to identify asymptomatic patients that only require active surveillance, as well as those with an advance disease, in a simple and inexpensive way. Patients are stratified into low-, intermediate- or high-risk subgroups in the Rai staging system according to the presence of lymphocytosis, anemia or thrombocytopenia, together with other clinical observations such as lymphadenopathy, splenomegaly or hepatomegaly. The Binet stages (A, B or C) also consider the parameters previously mentioned, in addition to the hemoglobin levels and platelet counts [1]. Subsequently, the lymphocyte doubling time and bone marrow infiltration were implemented as easily measurable prognostic factors [45,46].

#### 3.2.2. Serum Markers

In the late 1990s, serological tests allowed us to identify new prognostic factors that have been validated over years, such as Beta-2 microglobulin (β2M), thymidine kinase (TK) and LDH. β2M has been considered as an independent risk factor of progression-free survival (PFS) and overall survival (OS) [47,48], and a retrospective study demonstrated that lower β2M levels were independent predictors of complete remission after fludarabine-based chemoimmunotherapy (CIT) [49]. In a similar way, high levels of TK and LDH have been associated with shorter PFS [47,50,51,52].

Even though these scoring systems and serum markers are still used in clinical practice, they are not sufficient to elucidate the prognosis in the context of CLL heterogeneity. More recently, the development of new techniques and the improvement of molecular and genetic CLL characterization have raised a plethora of prognostic biomarkers that have been proven to be useful in patient risk stratification and therapy response prediction (Table 1) [53,54].

#### 3.2.3. *IGHV* Status and Stereotypes

In 1999, Hamblin et al. and Damle et al. determined at the same time the relevance of the mutational status of the immunoglobulin heavy-chain variable region (*IGHV*) in CLL [55,56], which has become a key prognostic factor extensively used in clinical practice [2,19]. According to the similarity to the germline sequence in CLL cells, patients with more than a 2% deviation in the *IGHV* region have mutated *IGHV* (*IGHV*-M), while patients with less than 2% are considered *IGHV*-unmutated (*IGHV*-UM). Those with *IGHV*-M have traditionally exhibited a good prognosis and have been associated with low-risk genetic alterations, while *IGHV*-UM patients have a more aggressive disease and are more likely to develop RS [57,58]. In addition to its value to predict the clinical outcome, the *IGHV* mutational status has been shown to be able to predict the treatment response (Table 1) [59]. Some studies proved that *IGHV*-M patients benefit from CIT, while *IGHV*-UM patients exhibit shorter PFS in response to these regimens [60,61].

*IGHV*-UM is associated with high-risk genetic lesions and with a more often stereotyped B-cell receptor immunoglobulin (BCR IG). The most common BCR IG stereotyped subsets in CLL are named #1, #2, #8 (associated with a bad prognosis) and #4 (associated with a good outcome) [62]. A recent study has identified subset #2 as an independent risk prognostic factor and could be considered for refining the stratification of CLL patients, especially in *IGHV*-M cases [62]. However, while the assessment of *IGHV* status is widely standardized and validated [63], stereotype identification is more complex and still restricted to research.

#### 3.2.4. Immunophenotypic Markers

In the early 2000s, the implementation of flow cytometry in clinical practice allowed to validate certain immunophenotypic markers as prognostic indicators, such as the expression of CD38 and ZAP70. CD38 positivity has been related to a shorter OS, and ZAP70 expression also constitutes a risk factor for the progression and development of RS [55,58,64]. Moreover, CD38 positivity (≥30%) and a high ZAP70 expression (≥20%) have been associated with *IGHV*-UM [65,66,67]. In the subsequent years, the search for other flow cytometric prognostic markers in CLL has uncovered CD49d [68], which is a more recent indicator of the disease progression also correlated with a poor outcome (Table 1) [69,70].

#### 3.2.5. Chromosomal Alterations

Chromosomal abnormalities are a hallmark of CLL. Since the 1990s, it has become evident that certain cytogenetic abnormalities have an impact on the clinical outcome of CLL patients [10,71,72]. However, the low mitotic rate obtained for CBA limited the assessment of the cytogenetic alterations [73]. The implementation of the FISH technique overcame this issue. Over the last 20 years, the FISH analysis has become the gold standard for cytogenetic risk stratification in CLL, allowing us to assess the most recurrent cytogenetic abnormalities with a prognosis impact in a more systematic manner [11].

Chromosomal alterations detected by FISH are present in over 80% of patients. At diagnosis and before the first therapy, the most common are deletions of 13q, followed by trisomy 12, and deletions of 11q and 17p. In the chemotherapy era, the presence of 13q deletion has been associated with a favorable outcome (median survival 133 months), similar to that of patients with a normal karyotype. Trisomy 12 contributes to an intermediate prognosis (median survival 114 months), while the 11q and 17p deletions (del(11q) and del(17p), respectively) have been related to worse outcomes (median survival 79 and 32 months, respectively) [11]. These findings have been subsequently validated in several studies, along with the increased risk associated with a high percentage of altered nuclei for each abnormality detected by FISH [74,75,76,77,78,79]. Nowadays, the classic four-probe CLL FISH panel is usually performed in routine clinical practice, being at least mandatory during the evaluation of del(17p) before starting any treatment due to its value not only as prognostic but, also, as predictive biomarker (Table 1) [20].

Other alterations, such as 14q rearrangements or the deletion of 6q, have also been considered as intermediate-risk cytogenetics, although they are not routinely incorporated in the CLL FISH panel for risk stratification [80,81,82,83,84]. The development of genomic array platforms has contributed to the identification of a huge amount of copy number alterations less frequent in CLL, being commonly found in the context of complex karyotypes [85,86,87,88,89,90]. However, these studies have been inconclusive with respect to the value of such higher resolution approaches for the risk assessment in CLL. Thanks to the introduction of modern cell stimulation protocols, conventional CBA has recovered its prognostic relevance in the last years, since recent reports have shown that complex karyotype contributes to an adverse outcome [91,92,93,94]. It has been described as a prognostic marker for refractoriness not only during chemoimmunotherapy [15,95,96] but, also, to TA [97,98]. Whether 3 or 5 is the appropriate cutoff for the number of abnormalities to define a complex karyotype is still debated [15,90]. Responding to these developments, the recently updated iwCLL guidelines state that CBA before treatment initiation is “desirable” in the context of clinical trials and also useful in general practice [20].

#### 3.2.6. TP53 and ATM Alterations

In the 2010s, additional molecular information at the mutational level was added to the FISH and cytogenetic analyses. The *TP53* gene, which is encompassed in del(17p), can be not only deleted in CLL but, also, recurrently mutated [99]. In fact, 70% of patients with del(17p) harbor mutations in the remaining allele, which results in a TP53-biallelic inactivation and dismal prognosis [100,101,102]. The assessment of the *TP53* status is crucial to predict the clinical outcome and therapy response, as its alterations contribute to a poor prognosis and chemotherapy resistance [103,104,105,106]. According to the recently published recommendations, mutational screening for the *TP53* gene should complement the FISH analysis for genetic risk stratification in CLL and the decisions before each therapy (Table 1) [2,20,107].

The deletion of 11q frequently encompasses the 11q23 region harboring the *ATM* gene, another tumor suppressor that is involved in the DNA damage response. In a similar way to del(17p)/*TP53* mutations, around 30% of 11q-deleted patients showed *ATM* mutations in the remaining allele, which may affect survival and response to chemotherapy [108,109,110]. Unlike the *TP53*-altered cases, a poor prognosis due to *ATM* alterations might be overcome by the administration of novel agents such as ibrutinib in treatment-naïve patients, as well as in CIT-relapsed/refractory patients [111,112,113,114]. Nonetheless, ibrutinib-relapsed/refractory patients with these alterations exhibit an inferior outcome, demonstrating the necessity of new combination therapies [115,116,117,118].

#### 3.2.7. Novel Gene Mutations and Clonal Evolution

In the last decade, the expansion of next-generation sequencing (NGS) has contributed to get a deep insight into the mechanisms of the pathogenesis of CLL [12,13,14,119,120,121,122]. Two whole-genome sequencing (WGS) and whole-exome sequencing (WES) studies, including more than 800 patients, demonstrated the vast genetic heterogeneity of CLL with the identification of more than 50 potential drivers [12,13], 29 of them commonly mutated in both studies [123]. These studies not only validated the presence of the recurrent mutations in *NOTCH1*, *SF3B1*, *ATM* or *TP53* [99,124,125,126,127] but also identified the highly frequented mutated genes such as *MYD88*, *POT1*, *CHD2*, *XPO1*, *BIRC3*, *FBXW7* and *DDX3X*, as well as the novel recurrent mutations in *RPS15*, *IKZF3*, *NFKBIE* or *EGR2* [128,129]. Several studies have demonstrated the prognostic impact of some of these genetic alterations in the time to first treatment (TTFT), PFS and OS. Specially, *NOTCH1*, *SF3B1*, *BIRC3* and *TP53* mutations have all been associated with *IGHV*-UM and an unfavorable prognosis (Table 1) [95,130,131,132,133,134,135].

NGS analyses have demonstrated that the increasing number of driver mutations has been also correlated with an inferior outcome [13,136,137,138]. In addition, the molecular characterization of large CLL cohorts has revealed patterns of co-occurrence or mutual exclusivity between genetic alterations that could also impact the clinical outcome [12,135]. Associations between trisomy 12 and *NOTCH1* mutations, as well as the deletion of 11q with *SF3B1* or *BIRC3* mutations, have been described, showing that these genetic mutations could further refine the prognosis of those cytogenetic subgroups [135,139,140,141,142,143,144,145]. Moreover, a recent study has shown that a subset of patients with a co-occurrence of 11q deletion and *TP53* alterations had a highly adverse outcome [117]. By contrast, the mutations in *MYD88* appeared in higher frequencies within 13q-deleted patients, being associated with a good prognosis and *IGHV* mutation [12,144,146].

NGS studies also demonstrated that clonal evolution contributes to the clinical variability in CLL patients [147,148,149]. These studies identified subclonal populations with a wide range of genetic mutations that could have a prognostic impact. These subclonal mutations could be present not only in B-mature cells but, also, in hematopoietic progenitors, and their variant allele frequencies could vary after the therapy administration or even without any treatment pressure [149,150,151,152,153]. In this context, the selection or appearance of certain mutations may determine the treatment response [12,154,155]. *TP53* is the main player of resistance to chemotherapy, since fludarabine-based regimens can exert a selection advantage for *TP53*-aberrant clones [115,147,156]. Apart from *TP53*, recent reports have provided evidence of an association between *NOTCH1* mutations and a lack of benefit of the anti-CD20 rituximab, suggesting that *NOTCH1* could have predictive potential (Table 1) [122,157,158,159]. In the era of targeted-therapies, the mutations in *BTK* and *PLCG2* have appeared in BCR-inhibitor refractory patients, as well as *BCL2* mutations in venetoclax-resistant CLLs (Table 1) (see Section 4 about treatment).

In light of the NGS findings, some studies have proposed the incorporation of these gene mutations in cytogenetic risk stratification [82,142,144,160] in order to refine the prognosis of patients in terms of the TTFT and OS. Besides, genetic profiling could also be extremely useful to predict the therapy response and to facilitate the decision-making for the treatment administration. However, the impact of some genetic alterations during prognosis need further validation, as the information for particular genes are contradictory, and new drivers are continually being discovered. In addition, these are high-cost and technically demanding approaches, and their implementation, as well as data analysis and interpretation, require expertise in the field [123]. For these reasons, NGS is a promising tool for improving CLL management and the prognostic score systems, but significant work for the optimization of the process and data harmonization is still needed.

#### 3.2.8. Noncoding Alterations and Epigenetics

MiRNAs are a small group of noncoding RNAs that play an important role in the regulation of gene expression. MiR-15a, and miR-16-1 located in 13q14, behave as tumor suppressors and were the first miRNAs used for outcome prediction in CLL [161,162,163]. Further studies have shown that miRNA profiling could contribute to refine the CLL prognosis. The expression levels of miR-155, miR-181b, miR-29a/b and miR-34a have been correlated to other prognostic biomarkers such *IGHV*, *TP53* status or ZAP70 expression, affecting the clinical outcome of CLL patients [164,165,166,167]. 

During the last decade, NGS studies have shown the presence of noncoding mutations in CLL patients [13,168]. In the case of *NOTCH1*, the noncoding mutations have the same clinical consequences as the coding ones [13,169].

Different methylation profiles have been associated with prognostic factors such as *IGHV* status or cytogenetic alterations [170,171,172,173]. Even patients with CLL can be grouped into three distinct epigenetic subclasses with different clinical features and outcomes [174,175]. Recently, chromatin remodeling can depend on the *IGHV* status and other genetic alterations (*MYD88* or trisomy 12) [176].

Despite all the previous findings, more studies are required to determine whether microRNA, noncoding and epigenetic profiles from CLL cells should be incorporated into clinical practice.

### 3.3. Risk Scoring Systems

**Figure 1:**
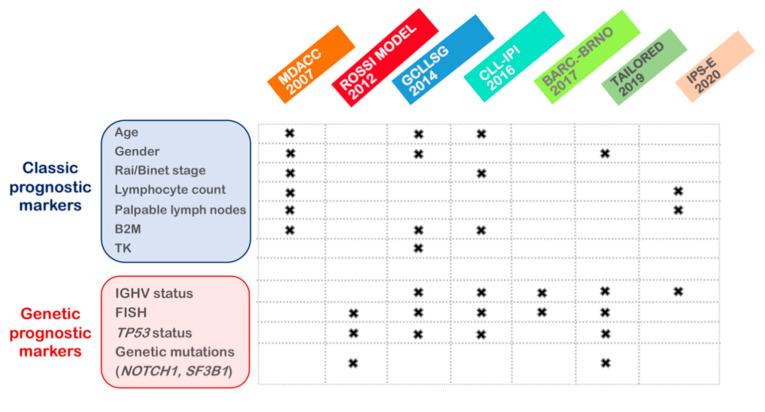
Prognostic models of CLL, including classic and genetic prognostic markers.

As commented on, several prognostic biomarkers have been identified during the last 30 years in order to better predict the clinical outcome of CLL patients. In the last years, many efforts have been made to reduce redundant prognostic information, resulting in the emergence of different prognostic models [177]. The Rai and Binet systems still remain as the backbone of prognostication due to their simplicity and low cost [1,43,44]. However, these staging systems have limited power to predict the evolution of the disease and response to therapy. Other prognostic score systems have subsequently implemented biological features, starting by serum markers such as β2M or TK (MDACC nomogram) [48] and followed by FISH/cytogenetic and *IGHV* information (GCLLSG model, Barcelona-Brno, CLL-IPI or IPS-E) [53,178,179,180,181,182] and also including genetic mutations (the Rossi model and the Tailored approach) [160,183] (Figure 1). Nonetheless, in the era of novel targeted therapies, treatments and prognostication are rapidly evolving, and validation of the traditional prognostic parameters, as well as the implementation of new indicators, is needed to ensure the optimal management of patients. In fact, some prognostic markers that were proven to be useful in the chemotherapy era lost their prognostic value in refractory/relapsed CLLs treated with TA. Conversely, other factors such as the achievement of minimal residual disease (MRD) negativity, have been shown to be an indicator of PFS and OS in the last years [184,185].

## 4. Treatment

### 4.1. Treatment Evolution on the Last Decades

Advances in the understanding of CLL biology have resulted in the development of new therapeutic approaches that have dramatically improved patient outcomes [186]. Recently, the identification of the specific therapeutic targets involved in the intracellular signaling pathways, such as the B-cell receptor (BCR) or BCL-2 (B-cell lymphoma), has revolutionized the treatment of CLL patients. CIT-based regiments were the standard of care for many years but have taken a backseat, with TA and their combinations occupying first place due to their excellent efficacies. The development of second-generation anti-CD20 molecules, in combination with targeted molecules, has also contributed to the changes in the therapeutic landscape. Although the majority of CLL patients with an active disease have benefited from this progress, probably those with major improvements in their quality of life and life expectancy have been elderly and/or high-risk patients [187]. However, the new treatment approaches also come with challenges, such as the emergence of drug resistance, toxic and adverse effects and treatment costs. Combination therapies, as well as the incorporation of other TA, will help to optimize the treatment approaches in the near future.

Other approaches, such as radiation therapy or splenectomy, have been abandoned in favor of CIT or TA, in most cases [188]. An exception in which these treatments might be considered is in a palliative setting. As CLL lymphocytes are radiation-sensitive, radiotherapy might be used in a palliative patient with compression symptoms [189]. A splenectomy might be effective for patients with massive splenomegaly refractory to other treatments [190]. Despite the great treatment evolution during the last years, it is important to point out that the majority of CLL patients are still monitored with a ‘watch and wait’ approach until the balance of risks and benefits favors the treatment initiation [191]. Indeed, a substantial fraction of CLL patients do not require CLL-related therapy during their lifetime [7].

#### 4.1.1. Chemoimmunotherapy

Over the past 50 years, and before the introduction of TA, the activity of the chemotherapy agents comprising alkylating agents (chlorambucil, cyclophosphamide and bendamustine); nucleoside analogs (fludarabine, pentostatin and cladribine) and corticosteroids was remarkable in patients with CLL. At the beginning, chlorambucil monotherapy was the therapeutic “gold standard” for several decades, but later, fludarabine-based regimens took advantage due to their superior overall response rates (ORR) compared with the other treatment regimens containing alkylating agents or corticosteroids [192].

In the early 2000s, the addition of anti-CD20 antibodies to chemotherapy resulted in prolonged survival, and CIT regimens therefore became the gold standard therapy. The combination of fludarabine, cyclophosphamide and rituximab (FCR) [193,194] was commonly used for younger, fit patients; bendamustine combined with rituximab (BR) [195,196,197] was commonly used for unfit patients and chlorambucil with anti-CD20 antibodies was used for elderly patients with coexisting conditions [198,199]. One of the potential risks of anti-CD20 antibodies is the reactivation of hepatitis B. Thus, virus B serologic testing is mandatory in all patients before anti-CD20 treatment initiation, and prophylactic antiviral therapy must be initiated before treatment in cases with a risk of reactivation. Another worrying issue associated with CIT is the long-term risk of inducing secondary neoplasia, including myelodysplastic syndromes and acute myeloid leukemia [61].

In the last years, some randomized clinical trials improved the survival and showed better side effect profiles with the TA [200,201]. Nowadays, the use of chemoimmunotherapy is steadily declining. An exception could be the group of young fit patients with *IGHV*-M, who often stay in remission for more than 10 years after treatment with the FCR regimen. For such patients, FCR treatment remains an alternative to the TA until we have a longer follow-up on ibrutinib-treated patients [61].

#### 4.1.2. Bruton Kinase Inhibitors

Ibrutinib is an oral small molecule acting as a Bruton tyrosine kinase inhibitor (BTKi). This drug is widely used nowadays not only as a frontline treatment but, also, in the relapse setting [113,202]. This is supported by the very satisfactory results recently shown in the phase 3 clinical trials RESONATE [203] and RESONATE-2 [204] (Table 2). Even though, in both trials, the control arm was not the best “standard of care”, their results were impressive, showing a high ORR and survival benefit in the ibrutinib arm, with a follow-up of more than 5 years, for all the CLL subgroups. The first results from RESONATE showed the excellent efficacy of ibrutinib in refractory/relapse (R/R) CLL patients, leading to Food and Drug Administration (FDA) approval in 2014 [205]. The second clinical trial experimented the use of ibrutinib as a frontline therapy [204]. More recently, ibrutinib was compared to CIT in treatment-naive CLL patients, questioning the need for CIT even in the subgroup of young, low-risk patients. The combination of ibrutinib–rituximab (IR) was superior to FCR in terms of the PFS and OS in ECOG-ACRIN E1912. This benefit was observed for all the analyzed subgroups, with the exception of *IGHV*-M patients, in which both treatments achieved similar results, and a long follow-up is required to determine the best option for this population [201]. For patients not able to tolerate FCR, the ALLIANCE trial compared BR to ibrutinib +/− rituximab. Patients receiving ibrutinib showed a longer PFS than patients treated with BR. Benefits in the OS have not been observed to date, with a median follow-up of 38 months. Furthermore, rituximab did not improve the PFS compared to patients treated with ibrutinib monotherapy [200].

Ibrutinib is not free from adverse events, with the most frequent being mild diarrhea, fatigue, nausea, bruising and arthralgia, while the most severe and less common are infections, atrial fibrillation, hypertension and ventricular arrhythmia [210]. Additionally, the data from real-life studies show that the major cause of discontinuation is off-target toxicity rather than progression [211]. Probably, a better selection of patients with cardiovascular comorbidities or at a high risk of bleeding or infection can optimize this discontinuation rate. On the other hand, potential benefits of ibrutinib include a modulating effect on the immune system [212].

Currently, second-generation BTKi are under investigation. These inhibitors join more selectively to their therapeutic target, improving their toxicity profile due to less frequency of the off-target events. Of them, acalabrutinib is the most mature, as the FDA has recently approved it for CLL patients (first-line and relapse) based on last year’s results of the ELEVATE-TN [206] and ASCEND [207] phase 3 clinical trials. Both trials demonstrated superiority in the acalabrutinib arms, with a good safety profile, as shown in Table 2. Moreover, the addition of obinutuzumab to acalabrutinib could provide a better PFS than acalabrutinib monotherapy in therapy-naive CLL patients but neutropenia in 30% of patients [206]. Zanubrutinib or tirabrutinib are the other second-generation BKTi under clinical development. Both have demonstrated encouraging activity in CLL patients, with a low incidence of off-target toxicity in their phase 1 and 2 studies [213,214]. Specifically designed to overcome the acquired resistance to ibrutinib, a new family of reversible BTKi emerged in 2020. These agents are now in the early phases of research and are soon to demonstrate their applicability in real life. Among them, fenebrutinib, LOXO 305 and ARQ 531 are under active clinical investigation nowadays [215].

#### 4.1.3. BCL-2 Inhibitors

Venetoclax is an oral BCL-2 inhibitor highly active in patients with CLL. The clinical development of this drug has lagged behind that of ibrutinib, although its effectiveness seems just as promising. The first clinical trials of venetoclax in patients with R/R CLL showed high response rates in terms of the PFS and ORR across all subgroups of CLL patients [216,217]. Based on the CLL-14 and MURANO phase 3 trials (Table 2) [208,209,218,219], venetoclax in combination with anti-CD20 has been recently approved for frontline treatment and treatment for relapsed CLL. As opposed to BCR pathway inhibitors, venetoclax induces deep remissions with high rates of MRD that allow treatment discontinuation. To date, venetoclax plus obinutuzumab has yielded the highest MRD-negative response rate in a randomized trial so far [220]. An extended follow-up of the CLL-14 and MURANO trials has recently been published, confirming the notorious clinical benefit of the combinations with venetoclax and demonstrating an OS benefit for R/R patients treated with venetoclax–rituximab. The rates of MRD negativity were also significantly higher in the venetoclax arm of both trials [208,218].

Venetoclax requires special measures (initial ramp-up escalation dose, vigorous hydration and laboratory test monitoring) to mitigate the risk of tumoral lysis syndrome (TLS) observed in the first clinical studies. Taking into account the aforementioned factors, TLS is not a big concern and has been reported in a low proportion of patients. In contrast, the most frequent grade ≥3 adverse event is neutropenia detected in around 50–60% of the patients, although not followed by a higher risk of infection [208,218].

#### 4.1.4. PI3K Inhibitors

Idelalisib is the first-in-class phosphatidyl-inositol 3-kinase inhibitor (PI3Ki) used in R/R CLL patients. Its clinical development was contemporary to ibrutinib, and it has been demonstrated to be an active oral small molecule, preferably used in combination with rituximab. This was shown in a phase 3 study that randomized 220 patients to receive rituximab plus idelalisib or a placebo. Patients receiving idelalisib significantly improved their PFS (19 vs. 6 months) and their OS (41 vs. 35 months), despite an extensive cross-over [221], and achieved a high rate of ORR. However, these benefits seem inferior to those obtained with BTKi, as was recently confirmed in the ASCEND clinical trial [207].

Toxicity has limited the use of idelalisib in real life, with a high rate of infectious (pneumonia) and autoimmune side effects (colitis, pneumonitis and hepatitis). Duvelisib is another PI3Ki granted by the FDA in 2018 for the treatment of R/R CLL patients after at least two prior therapies. It has also been demonstrated to be active in CLL, but, again, toxicity might limit its widespread use. Umbralisib is a next-generation PI3Ki with a much better toxicity profile, as it has been related to fewer immune-mediated toxicities or severe opportunistic infections to date [222]. Different clinical trials of umbralisib alone or in combination are ongoing and will help to elucidate its role in this rapidly changing treatment era.

#### 4.1.5. Immunotherapy

The surface antigen CD20 is the target of antibodies such as rituximab, ofatumumab and obinutuzumab, which are currently approved for CLL. These antibodies are commonly administered in combination with chemotherapy or targeted therapies. 

Recently, advances in monoclonal antibody technology have resulted in the development of new antibodies with improved therapeutic effectiveness. Ublituximab stands out, a next-generation CD20 antibody with encouraging results, especially in combination with the targeted molecules [222] or other monoclonal antibodies such as cirmtuzumab (anti-ROR 1), MOR00208 (anti-CD19) or otlertuzumab (anti-CD37). Less advanced are the bispecific antibodies and immunomodulatory antibodies [215].

Alemtuzumab, as anti-CD52 monoclonal antibody, is approved for the treatment of CLL. It was indicated, before the TA “era”, especially for patients with del(17p)/*TP53* mutations. However, the use of alemtuzumab is exceptional today due to the severe immunosuppression and the high rates of infectious complications associated with this drug [223]. Moxetumomab pasudotox, an antibody–drug conjugate targeting CD22 and delivering a cytotoxic agent simultaneously, has also been unsuccessful in treating CLL, unlike hairy cell leukemia. This is explained by the lower expression of CD22 in CLL lymphocytes [224].

#### 4.1.6. Combinations of Novel Agents

TA have changed the treatment landscape of CLL. Combinations of these targeted treatments with CIT, CD20 monoclonal antibodies and between them is what the immediate future holds. This approach aims to limit the toxicity, cost and resistance and achieve profound responses with MRD that can lead to the potential curation of the disease and treatment discontinuation. In particular, existing evidence indicates that anti-CD20 plays a synergistic role when used in combination with venetoclax. The combination of second-generation anti-CD20, such as obinutuzumab with BTKi, also appears to be beneficial [196,218,220,225].

Regarding the combinations between CIT and TA, probably the most interesting studies are those including young, fit, treatment naïve patients with mutated *IGHV*. Some of them have demonstrated very high rates of negative MRD, allowing the discontinuation of TA. Ultimately, combinations between TA with or without the addition of anti-CD20 have shown preliminary promising outcomes, with high rates of MRD negativity making possible treatment discontinuation as well. Nowadays, countless clinical trials are ongoing on this field. The most relevant combinations are summarized in Table 3 [217,218,220,226,227,228,229,230,231,232,233,234].

Moreover, three ongoing, independent phase 3 trials stand out (ECOG-ACRIN EA9161 for young patients, ALLIANCE A041702 for patients >70 years old and CLL-17 (fit and unfit patients)), exploring different combinations with venetoclax, ibrutinib and obinutuzumab that allow treatment disruption in some of their arms. The results of these studies will probably again change routine practices in the near future.

#### 4.1.7. Cellular Therapy

Allogeneic stem cell transplantation (allo-TPH) is a potentially curative approach to CLL patients. Years ago, it was indicated in patients with poor prognostic factors (early relapses, refractory to fludarabine or harboring TP53 abnormalities). TA have changed the natural history of CLL, and therefore, the role of allo-TPH in this new era is less clear. A recently published retrospective study reported the outcome of 65 patients undergoing allo-TPH after at least one TA, pointing out that it is a viable long-term disease control strategy. In this study, the investigators observed that PFS was predicted by the hematopoietic cell transplantation-specific comorbidity index. No differences were observed among the patients receiving previous TA (one or two ibrutinib/venetoclax) or TA and CIT as the previous treatment [235]. Currently, most guidelines recommend it for patients with high-risk CLL that have relapsed or are refractory to at least one TA or in cases of clonally related Richter transformation with a response to chemotherapy [202,236]. However, some questions, such as the optimal timing of the procedure, remain unanswered.

CLL was a pioneering disease in which chimeric antigenic receptor T (CAR-T) cells targeting CD19 were tested [237], but the ORRs were not as good as those observed in other diseases, and the estimated PFS at 18 months was around 28% [238]. In order to optimize its applicability in CLL, different strategies are under investigation, such as those using ibrutinib concurrently with CD19 CAR T cells [239]. With this approach, the ORR and PFS were improved, and a better toxicity profile was observed after one year of follow-up. Even so, these results need more robustness to be adopted in clinical practice. Another option under study is the use of modified cord blood natural killer cells to express anti-CD19 CAR [240].

### 4.2. Current Treatment Strategies

**Figure 2:**
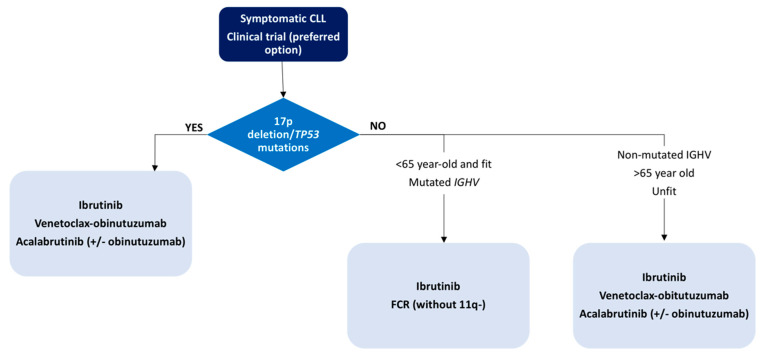
First-line treatment algorithm for CLL patients.

**Figure 3:**
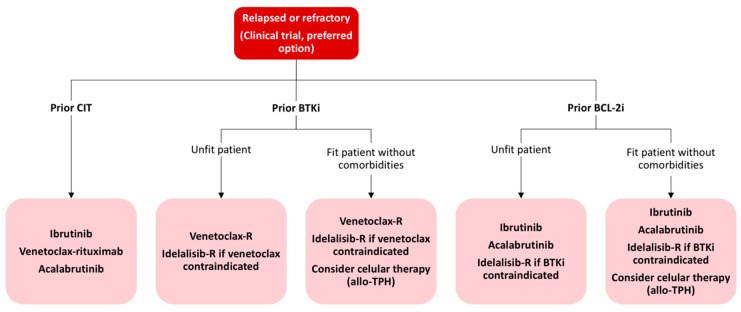
Treatment algorithm for relapsed or refractory CLL patients. CIT: chemoimmunotherapy; BTKi: Bruton tyrosine kinase inhibitor; R: rituximab; allo-TPH: allogeneic stem cell transplantation; BCL-2i: BLC2 inhibitor.

In contrast to the treatment paradigm shift previously described, the treatment indications remain without changes, as outlined by the consensus guidelines published by the iwCLL in 2018 [20]. For the time being, asymptomatic patients must be monitored without active treatment irrespective of the risk, even though some studies treating high-risk asymptomatic patients are ongoing, aiming to answer if this approach is beneficial [241]. With the existing evidence and actual approval, we propose a treatment algorithm based on patient age, comorbidities and genetic abnormalities, as depicted in Figure 2 and Figure 3.

### 4.3. Drug Resistance

Despite the significant clinical efficacy in most CLL patients treated with TA, in some of them, the treatment fails. The number of patients who progress or develop clinical resistance is expected to increase in the following years due to the increasing number of patients treated with TA and the long-term administration of these agents. Thus, understanding the potential resistance mechanisms will help to design new treatment strategies to prevent resistance and avoid relapse.

#### 4.3.1. Ibrutinib Resistance

While ibrutinib is an effective therapy leading to durable responses, some patients acquire resistance and relapse [242]. In 2014, a study using whole-exome sequencing discovered acquired mutations within the *BTK* gene in CLL patients relapsing on ibrutinib [243]. Further studies confirmed the presence of a *BTK* mutation in the CLL patients relapsing on ibrutinib [115,244,245], C481S being the most common mutation at the position of the binding site of the drug [246,247]. *BTK* mutations can be explained by the mechanism of action of ibrutinib, which binds to BTK with an irreversible covalent bond at position C481S. From there, ibrutinib inhibits the proliferative and antiapoptotic signals that are abnormally stimulated in CLL cells through the NF-κB pathway downstream a wide variety of signal transducers, including PLCG2, SYK or LYN, among others [248]. 

The second-most frequent mutations found in CLL patients who fail on ibrutinib treatment are *PLCG2* mutations [249]. The *PLCG2* gene encodes Cγ2, the protein immediately downstream of BTK, and its mutations mostly have an activating effect, resulting in continuous BCR signaling independent of the BTK activity [243,249]. 

The main characteristics and differences of the *BTK* and *PLCG2* mutations are summarized in Table 4. The acquired mutations in these genes have been detected in 80% of patients with ibrutinib failure and CLL progression. Resistance usually develops between the second and fourth year of ibrutinib treatment, but *BTK* and *PLCG2* mutations might be detected at low allelic frequencies up to 9–15 months before CLL progression [115,244]. In contrast to CLL progression on ibrutinib, which tends to occur later in therapy (after 12 months of attaining a response), Richter transformations mostly occur during the first 1 to 2 years of treatment [250,251].

**Figure 4:**
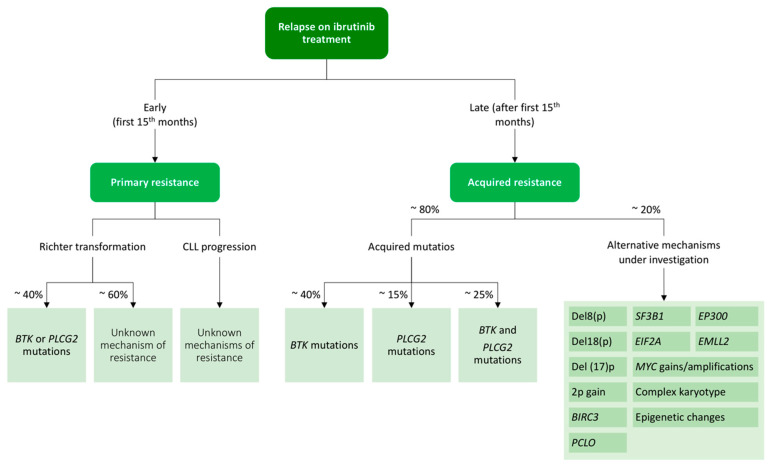
Resistance mechanisms to ibrutinib treatment in chronic lymphocytic leukemia.

On the other hand, there are approximately 20% of patients in whom *BTK* and *PLCG2* mutations cannot be identified. For them, alternative mechanisms of resistance such as 8p deletion or additional driver mutations have been described and are shown in Figure 4 [252].

Intrinsic resistance to ibrutinib is extremely rare and, conversely, has not been studied in depth. Three independent studies analyzed pretreatment samples from patients who relapsed on ibrutinib treatment and failed to find mutations at that moment. In patients that relapse early (first fifteen months), it is necessary to rule out a transformation into a high-grade lymphoma [242,253].

Different strategies have been suggested to overcome ibrutinib resistance, highlighting the use of TA targeting other pathways such as PI3K or BCL-2 or the use of reversible BTK inhibitors [253].

#### 4.3.2. Venetoclax Resistance

The resistance mechanisms of venetoclax are not as well-defined as those occurring after ibrutinib failure. This could be due not only to the later development of the drug but, also, to the implication of different independent molecular mechanisms. Similar to what happens with BTK mutations, a mutation at the G101V in *BCL2* has been implicated in the reduction of venetoclax binding to BCL2. This mutation was found in almost half of patients that progressed under venetoclax in a recent study of a small cohort of cases in 2019. Another mutation in BCL2, D103Y, has been also associated with venetoclax [254]. This and other mutations could coexist in same patients but as independent clones with different growth dynamics [254,255]. *BCL2* mutations were identified several months prior to clinical relapse (~25) [256] (Table 4).

Besides *BCL2* point mutations, other candidate resistance-associated aberrations have been reported, including mutations in the antiproliferative *BTG1* gene, aberrations of *CDKN2A/B*, the overexpression of *MCL1* and *BCL-XL* (pro-survival proteins) and the amplification of *AMP-1*, which can affect the OXPHOS pathway in mitochondria [155,256,257].

### 4.4. COVID-19 and CLL Treatment

The COVID-19 pandemic complicates the current clinical practice for CLL patients, making it more challenging. CLL patients are a population particularly susceptible to SARS-CoV-2 infection, with a high fatality rate (~32–34%) [258,259,260]. This is not surprisingly, as many of these patients harbor high-risk factors for developing severe COVID-19 (age, comorbidities and immunodeficiency) [261]. 

A recent study noted that ibrutinib may have a lung-protective effect and may attenuate inflammatory responses due to its inhibitory tyrosine kinase mechanism of action [262]. Thus, there is much debate on whether patients under BTKi should discontinue treatment if they contract the virus. Evidence is controversial and comes from case reports and a European retrospective study in which patients treated with ibrutinib had a better hospitalization rate [259,263,264]. On the other hand, an American retrospective study did not find this protective effective, even though most cases discontinued BTKi treatment after being infected with SARS-CoV-2. In addition, the second-generation BTKi acalabrutinib was used in a retrospective cohort of 19 severe COVID-19 patients without CLL, with encouraging results [265]. Hopefully, ongoing prospective clinical trials will clarify if targeting inflammation with a BTKi is a good strategy for COVID-19. In the meanwhile, expert recommendations advocate to limit the patient´s exposure to potential nosocomial SARS-CoV-2 and hold therapy until after recovery of the infection [266]. If the decision is to continue treatment with BTKi, special care must be taken towards the medical interactions and the hemorrhagic risk, as most critical patients are under an anticoagulant treatment in this phase of the disease.

## 5. Current Challenges

The impressive progress achieved in all fields of CLL (diagnosis, prognosis and treatment) in the recent years goes hand-in-hand with the emergence of new challenges. Some of them are listed below:

- How valid are the prognostic scores in the era of TA?
- Will high-risk early-stage patients benefit from early treatment?
- What are the practical implications of complex karyotype for treatment selection?
- Which are the subclones responsible for disease evolution, and how do they acquire an expansion benefit?
- What is the meaning of low-burden clonal and subclonal mutations?
- Is there still a role for CIT alone or in combination with TA in frontline young, mutated CLL patients?
- Will TA combinations be the best treatment option as the first-line therapy for all patients?
- What is the optimal sequence of TA in the treatment of CLL patients?
- What are the best options to overcome an acquired resistance with TA?
- Will the acquired resistant mutations detected early evolve into an overt resistance in all patients?
- What is the optimal timing for allo-TPH and/or CAR-T cells?
- Should patients with targeted therapies discontinue treatment if they get a SARS-CoV-2 infection?

The near future will certainly clarify some of the controversies, but we will probably end up with new open-ended questions that require investigation.

## Figures and Tables

**Table 1:** Clinical significance of the main prognostic markers in CLL.

Biomarkers	Clinical Significance in Prognosis
Rai/Binet advance stage	Associated with unfavorable disease course. Not enough to predict disease progression.
β2M high (>3.5 mg/L)	Predicts worse outcome and short-term remission after fludarabine-based CIT. Included in different risk scoring systems.
CD49d expression	Predicts shorter survival and remains valid for predicting treatment-free survival after ibrutinib treatment ^1^.
*IGHV* unmutated	Associated with a shorter time to first treatment and poorer response to CIT. Its assessment is highly recommended in pre-treatment evaluation and only once since its status remains stable during disease course.
Del(11q)/*ATM* mutation	Associated with a shorter time to first treatment but better response to BTK inhibitors in the presence of del(11q) ^1^.
Del(17p)/*TP53* mutation	Confers resistance to CIT and predicts rapid disease progression. Its assessment is mandatory in pre-treatment evaluation.
Complex karyotype	Predicts unfavorable outcome after CIT independently of *TP53* alterations. Its role is controversial after novel targeted agents ^1^.
*NOTCH1* mutation	Refines cytogenetic-risk stratification and is associated with worse outcome and poor response to rituximab treatment ^1^.
*SF3B1* mutation	Refines cytogenetic-risk stratification and has been associated with poor prognostis ^1.^
*BTK/PCLG2* mutation	Confers resistance to BTK inhibitors.
*BCL2* mutation	Confers resistance to venetoclax.
MRD positive	Predicts shorter progression free-survival for CIT. Remains valid for venetoclax-based regimens ^1^.

^1^ Not yet established prospectively. CIT: chemoimmunotherapy.

**Table 2:** Efficacy and safety of the most relevant new oral targeted therapy phase 3 trials.

Drug	Line	Trial	Treatment (N)	ORR	PFS	OS	AE ≥ G3	Follow Up	References
Ibru	1	Resonate-2	Ibru (136)	92%	NR	NR	Neutropenia (13%), pneumonia (12%), major hemorrhage (11%), hypertension (8%), anemia (7%), atrial fibrillation (5%), diarrhea (4%)	57 m	[204]
			Chl (133)	37%	15 m	NR			
	R/R	Resonate	Ibru (195)	91%	44.1 m	67.7	Neutropenia (25%), pneumonia (21%), hypertension (9%), major hemorrhage (10%) anemia (9%), atrial fibrillation (6%), diarrhea (7%)	65 m	[203]
			Ofatumumab (196)	24%	8.1 m	65.1			
Acala	1	Elevate-TN	Acala-Obi (179)	94%	NR	NR	Neutropenia (30%), pneumonia (6%), anemia (6%), atrial fibrillation (4%), diarrhea (4%), hypertension (3%), major hemorrhage (2%), infusion reaction (2.2%)	28 m	[206]
			Acala (179)	86%	NR	NR	Neutropenia (9%), anemia (7%), atrial fibrillation (3%), pneumonia (2%), hypertension (2%), major hemorrhage (2%), diarrhea (1%)		
			Chl-Obi (177)	79%	22.6 m	NR	Neutropenia (41.4%), thrombocytopenia (11.8%), anemia (7.1%), infusion reaction (5.6%), pneumonia (1.8%)		
	R/R	ASCEND	Acala (155)	81%	NR	NR	Neutropenia (15%), anemia (11%), pneumonia (5%), atrial fibrillation (5%), hypertension (2%), diarrhea (1%), major hemorrhage (1%)	16.1 m	[207]
			I.C.: Idela-R (119)	75%	15.8 m	NR	Neutropenia (39%), diarrhea (24%), pneumonia (8%), anemia (7%)		
			I.C.: BR (36)		16.9 m	NR	Neutropenia (31%), anemia (9%) pneumonia (3%)		
Ven	1	CLL-14	Ven-Obi (216)	85%	NR	NR	Neutropenia (53%), infusion reaction (9%), thrombocytopenia (9%), anemia (9%), pneumonia (7%), tumor lysis syndrome (2%)	39.6 m	[208]
			Chl-Obi (216)	71%	35.6 m	NR	Neutropenia (46%), thrombocytopenia (15%), infusion reaction (11%), anemia (7%), pneumonia (5%), tumor lysis syndrome (3%)		
	R/R	Murano	Ven-R (195)	92.3%	53.6 m	83.5% ^1^	Neutropenia (57.5%), infections (17.5%), anemia (10.8%), pneumonia (5.2%), tumor lysis syndrome (3.1%)	59.2 m	[209]
			BR (194)	72.3%	17 m	66.8% ^1^	Neutropenia (38.8%), infections (21.8%), anemia (13.8%), pneumonia (8%)		

^1^ Four-year PFS and OS; N: number; ORR: overall response rate; PFS: progression-free survival; OS: overall survival; AE: adverse event; G3: grade 3; R/R: relapsed or refractory; 1: first-line; NR not reached; m: months; Ibru: ibrutinib; Chl: chlorambucil; Acala: acalabrutinib; Obi: Obinutuzumab; Ofatu: Ofatumumab; Idela: idelalisib; Ven: venetoclax; R: rituximab; I.C.: investigator criteria; B: bendamustine.

**Table 3:** Trials using new combinations of novel agents with anti-CD20, chemoimmunotherapy and between them for CLL.

Therapeutic Approach	Treatment	Phase	N R/R	N TN	Duration of Treatment	Response Rate	% uMRD (BM)	References
TA + anti CD20	Ibru-R	II	208	27	Indefinite	92.3%	BM, 24 m: 19.8%	[226]
	Ibru					92.3%	BM, 24 m: 12.2%	
	Ibru-O (benda)	II	30	31	Possible if MRD-neg	100%	PB, 12 m: 48%	[230]
	Acala-O	Ib/II	26	19	Indefinite	92% (R/R) 95% (TN)	12 m: 15% (R/R), 26% (TN)	[231]
	Ven-R	III	389		24 months	92.3%	PB, 24 m: 62.4%	[217]
	Benda-R					72.3%	PB, 24 m: 13.3%	
	Ven-O	III		432	12 cycles	85%	BM, 12 m: 57%	[220]
	Chl-O					71%	BM, 12 m: 17%	
TA + CIT	FCR-ibru	II		85	Possible if MRD-neg	96%	BM, 24 m: 78%	[233]
	FCO-ibru	II		45	Possible if MRD-neg	73%	BM, 12 m: 100%	[227]
	FCR-duvelisib	Ib/II		32	24 months	88%	BM, 66% at best response	[232]
TA + TA	Ibru-ven	II	53		Possible if MRD-neg	89%	BM, 12 Mm 36%	[228]
	Ibru-ven	II		80	Possible if MRD-neg	88%	BM, 12 m: 61%	[234]
	Ibru-ven-O	Ib	25	25	14 cycles	88% (R/R) 84% TN	BM and PB, 7 m: 70%	[229]

TA: targeted agent; R/R: relapsed/refractory; TN: treatment-naïve; uMRD: undetectable minimal residual disease; BM: bone marrow; m: months; Ibru: ibrutinib; R: rituximab; O: Obinutuzumab; Acala: acalabrutinib; Benda: bendamustine; Ven: venetoclax; Chl: chlorambucil FCR: fludarabine, cyclophosphamide and rituximab; FCO: fludarabine, cyclophosphamide and Obinutuzumab; neg: negative; PB: peripheral blood.

**Table 4:** Acquired mutations observed in patients that become resistant to ibrutinib and venetoclax.

	Ibrutinib		Venetoclax
Mutation Type	*BTK*	*PLCG2*	*BCL-2*
Prevalence in relapsed patients	57%	13%	47%
Mechanism	Loss of covalent binding of ibrutinib to BTK	Activating BCR signaling independent of BTK	Disruption of the bond of venetoclax to BCL-2
Variants			
More frequent	C481S	Different subclones coexist with low allelic burden	G101V (subclonal)
Others	C481R, C281F, C481Y, R28S, G164D, T316A, T474I/S, R490H, Q516K, L528W, V537I	F82S, P664S, R665W, S707Y, S707P, S707F, L845F, L845V, L845G, L848R, D993Y, D993H, D1140N, M1141K, M1141R, S1192G	D103Y, A103T, A103G, A103V, A113G, A129L, V156A
Median time since drug exposure	34.3 months (14–76.8)	35.1 months (17.4–64.6)	36 months (6.5–73)

BTK: Bruton tyrosine kinase; PLCG2: phospholypaseCɣ-2; BCR: B-cell receptor.

## Data Availability

Not applicable.
